# Isoginkgetin Induces Caspase Cascade Activation and Cell Apoptosis via JNK Signaling in Oral Cancer

**DOI:** 10.7150/jca.123992

**Published:** 2025-11-14

**Authors:** Wei-En Yang, Chun-Yi Chuang, Chiao-Wen Lin, Chun-Wen Su, Meng-Ying Tsai, Shih-Chi Su, Heng-Hsiung Wu, Shun-Fa Yang, Yi-Tzu Chen

**Affiliations:** 1 Institute of Medicine, Chung Shan Medical University, Taichung, Taiwan.; 2 Department of Medical Research, Chung Shan Medical University Hospital, Taichung, Taiwan.; 3 School of Medicine, Chung Shan Medical University, Taichung, Taiwan.; 4 Department of Otolaryngology, Chung Shan Medical University Hospital, Taichung, Taiwan.; 5 Institute of Oral Sciences, Chung Shan Medical University, Taichung, Taiwan.; 6 Department of Dentistry, Chung Shan Medical University Hospital, Taichung, Taiwan.; 7 Whole-Genome Research Core Laboratory of Human Diseases, Chang Gung Memorial Hospital, Keelung, Taiwan.; 8 Department of Medical Biotechnology and Laboratory Science, College of Medicine, Chang Gung University, Taoyuan, Taiwan.; 9 Program for Cancer Biology and Drug Discovery, China Medical University, Taichung, Taiwan.; 10 School of Dentistry, Chung Shan Medical University, Taichung, Taiwan.

**Keywords:** oral squamous cell carcinoma, isoginkgetin, apoptosis, caspase, JNK

## Abstract

Isoginkgetin (IGG), a naturally occurring biflavonoid found in the leaves of many medicinal plants, is known to inhibit pre-mRNA splicing and display anti-cancer characteristics. However, knowledge regarding the use of IGG on oral squamous cell carcinoma (OSCC) lags behind that on the other common malignancies. The aim of this study is to explore whether IGG hinders OSCC proliferation and further investigated its oncostatic actions. We demonstrated that exposure of OSCC cell lines (HSC-3 and SCC-9) to IGG significantly diminished cell viability and induced apoptotic cell death. Furthermore, levels of several tentative apoptosis suppressors (cIAP-1 and XIAP) were decreased in IGG-treated HSC-3 and SCC-9 cells, accompanied with increased cleavage of caspases. Of note, such activation of caspase cascades by IGG was reduced by pharmaceutical inhibition of c-Jun N-terminal kinase (JNK) via a specific kinase antagonist, suggesting a functional connection of JNK activity with caspase activation during IGG-induced oral cancer cell apoptosis. In conclusion, we exhibited that IGG hampered cell viability and stimulated apoptotic events in OSCC, driven by a JNK-dependent pathway of caspase activations. Our findings present new insights into applications of a natural biflavonoid compound in fighting oral carcinogenesis.

## Introduction

Oral squamous cell carcinoma (OSCC) is the most common type of oral cancer, representing approximately 90% of oral malignancies 1. In patients with OSCC, surgery in combination with radiotherapy or chemotherapy are the primary treatment of choice. In addition, the use of an antagonist for the epidermal growth factor receptor (EGFR) has improved disease outcomes in OSCC patients receiving radiotherapy 2. Besides, immune checkpoint inhibitors that restore anti-cancer immunity have been demonstrated to extend patients' survival as combined with chemotherapy or radiotherapy 3-5. Yet, these treatment strategies did not significantly leverage the survival rate (roughly 50%) 6, mainly due to tumor recurrence and metastasis. This emphasizes the imperative to investigate complementary therapeutic modalities that can tackle these challenges. In this regard, the exploration of phytoconstituents that manifest favorable responses but minimal undesirable effects for the management of OSCC has come to the fore as a pivotal filed of research.

Isoginkgetin (IGG), originally extracted from the leaves of *Metasequoia glyptostroboides* (Dawn redwood, family: *Taxodiaceae*), is a naturally occurring biflavonoid shown to suppress cell invasion and migration by inhibiting the production of the matrix metalloproteinase 9 (MMP-9) 7, 8. Pharmacological investigations revealed that IGG functions as an effective inhibitor for transcription elongation 9 and pre-mRNA splicing 10, to some degree providing the mechanistic basis of its anti-cancer activity. Such inhibition of the splicing machinery by IGG in cancer cells enhanced the antigen presentation by MHC class I 11, thereby facilitating the adaptive immune response against tumor antigens 12. In addition, IGG was found to disturb protein homeostasis, eventually leading to cancer cell death 13. Intriguingly, IGG may induce cytotoxic autophagy in hepatocellular carcinoma via directly binding to the N terminus of cyclin-dependent kinase 6 (CDK6) and promoting its subsequent degradation 14. Although these *in vitro* and *in vivo* results have unveiled distinct anti-cancer features of this natural biflavonoid, beneficial effects of IGG on oral tumorigenesis are mostly unknown. In this study, we aimed to investigate whether IGG hampers OSCC progression and further explored the underlying mechanisms. Our findings highlight potential avenues for the use of a natural compound in the management of OSCC.

## Materials and methods

### Cell culture and reagents

Two cell lines of OSCC, SCC-9 and HSC-3, were obtained from the American Type Culture Collection (Manassas, VA, USA) and propagated in MEM medium (Life Technologies, Grand Island, NY, USA) as described previously 15. SCC-9 is derived from a human tongue squamous cell carcinoma and HSC-3, also originating from tongue carcinoma, is known for its highly aggressive behavior. Isoginkgetin (IGG) of HPLC grade with ≥ 98% purity was commercially acquired from Sigma-Aldrich. U0126 and JNK-IN-8 were purchased from Sigma-Aldrich (St. Louis, MO, USA), and SB203580 was obtained from Cell Signaling Technology (Danvers, MA, USA).

### Assessment of cell viability

Viability of OSCC cells in response to IGG was measured with a MTT (3-(4,5-dimethylthiazol-2-yl)-2,5-diphenyltetrazolium bromide) colorimetric assay (Sigma-Aldrich) as described previously 16. In brief, cells were cultured in the presence of IGG at various concentrations for 24 hr and assessed for cell viability by using MTT. Levels of cell proliferation/viability were evaluated according to the chemical yield of formazan following solubilization with isopropanol, which was spectrophotometrically measured at 563 nm.

### Flow cytometric analysis

Apoptotic cell populations were analyzed by monitoring the levels of annexin V flipping via flow cytometry as previously described 17. In brief, cells exposed to various concentrations of IGG for 24 hr were assessed for the levels of annexin V on the outer leaflet of the plasma membrane with an FITC-labeled Annexin-V/PI Apoptosis Detection kit (BD Biosciences, San Jose, CA, USA). The proportions of annexin V- or propidium iodide (PI)-positive cells were evaluated by using flow cytometry (Accuri C6 Plus flow cytometer, BD Biosciences, San Diego, CA, USA).

### Profiling of apoptotic proteome

Apoptosis-related protein markers in IGG-treated OSCC cells were profiled through a Proteome Profiler Human Apoptosis Array Kit (R&D Systems, Minneapolis, MN, USA) 18. This membrane-based antibody array allows the simultaneous detection of 35 human apoptosis-related proteins, including both pro-apoptotic and anti-apoptotic factors. Protein lysates of OSCC cell lines treated with and without IGG were collected and applied to the protein array analysis according to manufacturer's instructions. Pixel density for apoptotic markers was measured and normalized to that of reference array spots.

### Western blot

Protein lysates of cells under various conditions were harvested and subjected to SDS-PAGE assays. Individual protein targets were detected via a series of specific primary antibodies. These include Anti-cleaved Caspase-3 (ab2302), Anti-cleaved Caspase-8 (ab25901), Anti-pro-caspase-3 (ab32150), Anti-pro-caspase-8 (ab108333), and Anti-β-actin (ab8226) antibodies from Abcam (Cambridge, UK), Anti-Caspase-9 (#9502), Anti-cleaved Caspase-9 (#9505), Anti-PARP (#9542), Anti-Phospho-Erk1/2 (#4370), Anti-Erk1/2 (#9102), and Anti-c-IAP1 (#7065) antibodies from Cell Signaling Technology (Danvers, MA, USA), as well as Anti-Phospho-JNK (sc-6254), Anti-JNK (sc-7345), Anti-phospho-p38 (sc-166182), Anti-p38 (sc-7972), and Anti-XIAP (sc-55550) antibodies from Santa Cruz Biotechnology (Dallas, TX, USA). Visualization was conducted by hybridization with HRP-conjugated secondary antibodies (Dako Corporation, Carpinteria, CA, USA). Densitometry of immunoblots was analyzed via the ImageJ software.

### Immunofluorescence

Cells were grown on coverslips, treated with IGG for 24 hr, fixed, and permeabilized. Cell cultures were stained with a primary antibody against cleaved caspase 3 (#9661, Cell Signaling Technology, Danvers, MA, USA), visualized by hybridization with a fluorescence-labeled secondary antibody (#4412, Cell Signaling Technology), washed, fixed, and mounted in Fluoromount-G (Electron Microscopy Sciences) 19. Images were acquired by Olympus IX73 inverted fluorescence microscope using cellSENS microscope imaging software.

### Statistical analysis

Data represent mean ± standard deviation (SD) from at least two separate experiments. Significant difference was based on a *p* value of < 0.05 by Student's *t*-test.

## Results

### IGG stimulates cytotoxicity and apoptosis in OSCC cells

**Figure 1:**
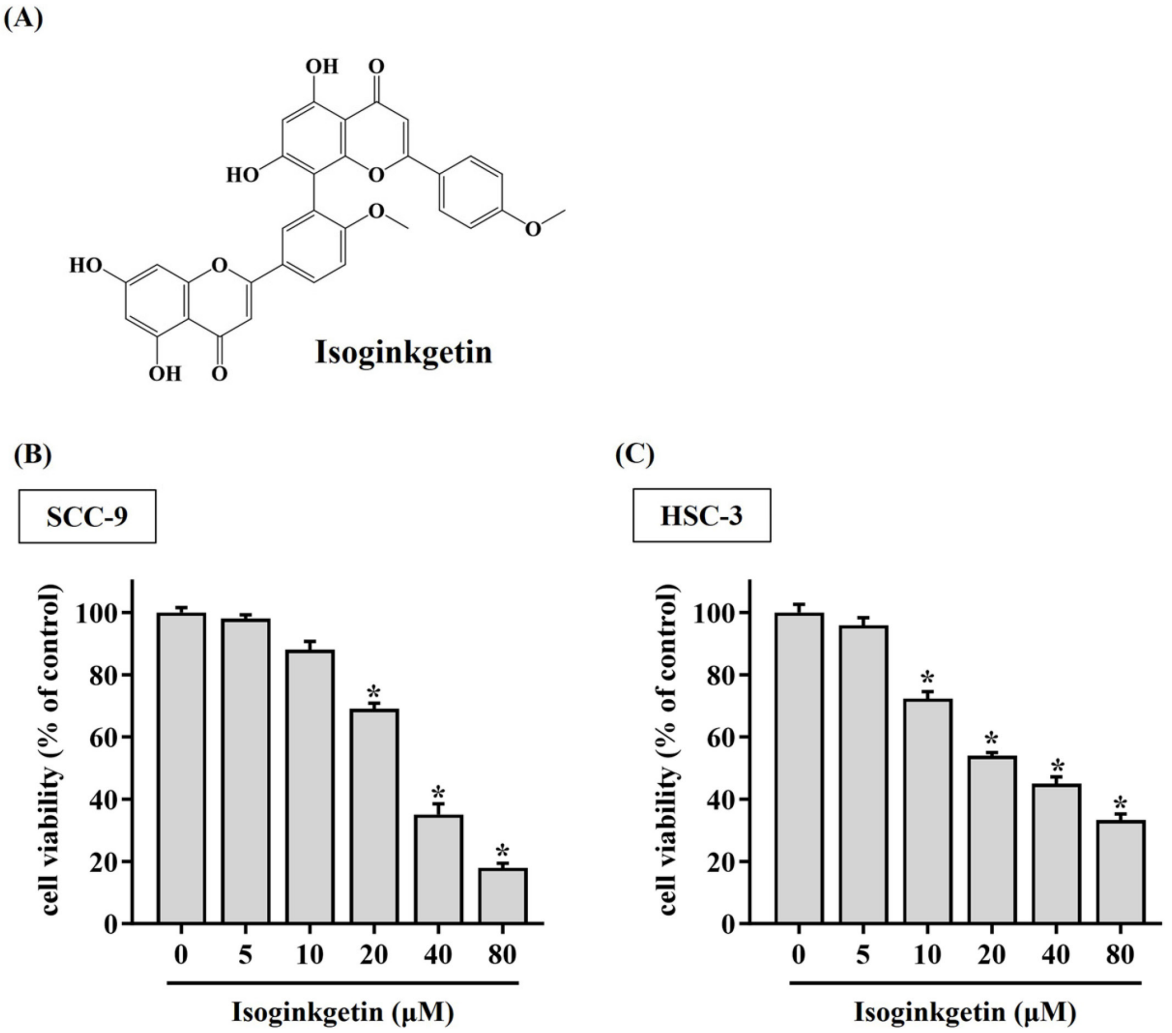
** IGG triggers cytotoxicity to OSCC cell lines.** (**A**) Structural formula of isoginkgetin (IGG). (**B-C**) IGG is cytotoxic to OSCC cells. SCC-9 **(B)** and HSC-3 cells **(C)** were cultured in the presence of indicated concentrations of IGG for 24 hr and evaluated for cell viability. Data represent the average ± SD from three separate experiments. *p < 0.05, in comparison with untreated cells using Students t-test.

**Figure 2:**
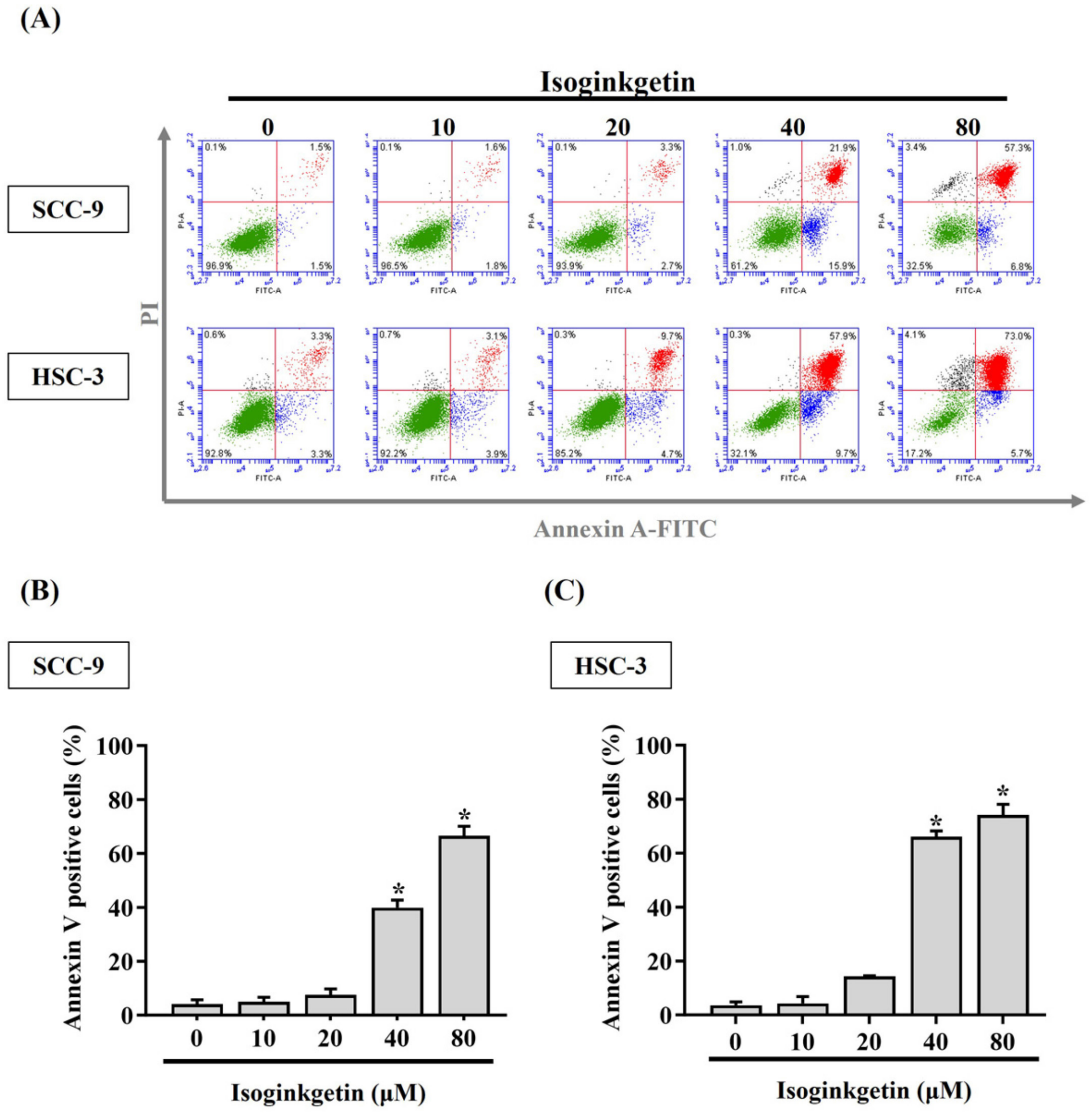
** IGG triggers apoptotic events in OSCC.** (**A**) SCC-9 and HSC-3 cells were maintained in the presence of indicated concentrations of IGG (10-80 μM) for 24 hr, stained with PI and annexin V, and assessed for apoptotic cell death by flow cytometry. Data are representative of three independent experiments. (**B-C**) Comparison of apoptotic responses among IGG-treated SCC-9 **(B)** and HSC-3 cells **(C)**. The proportion of annexin V-positive cells was measured, and data represent the average ± SD from three independent experiments. *p < 0.05, in comparison with untreated cells using Students t-test.

To clarify the potential of IGG on affecting OSCC progression, the viability of SCC-9 and HSC-3 cells treated with various concentrations of IGG (5 to 80 μM) was tested. We observed a reduction in cell viability of OSCC cells in response to 10 μM of IGG (**Figure 1**). Such cytotoxic effect appeared to be dose-dependent, as 40 and 80 μM of IGG profoundly interfered with cell proliferation of SCC-9 and HSC-3. This finding is in accordance with its anti-cancer properties noted in other tumor types 7, 12-14, unveiling a suppressive effect of IGG on oral carcinogenesis. Since a dose-dependent effect of IGG on influencing OSCC proliferation was demonstrated, we next examined whether IGG alters apoptotic responses in oral cancer. By monitoring the levels of annexin V flipping from the inner side to the outer leaflet of the plasma membrane, an increased proportion of apoptotic cell populations under the treatment of 40 and 80 μM IGG was detected in both cell lines (**Figures 2A-C**), revealing an elevation in OSCC apoptosis by IGG. These data suggest that the anti-cancer potential of IGG on oral tumorigenesis is attributed to induction of cytotoxicity and apoptosis.

### IGG reshapes apoptosis-related proteome in OSCC

**Figure 3:**
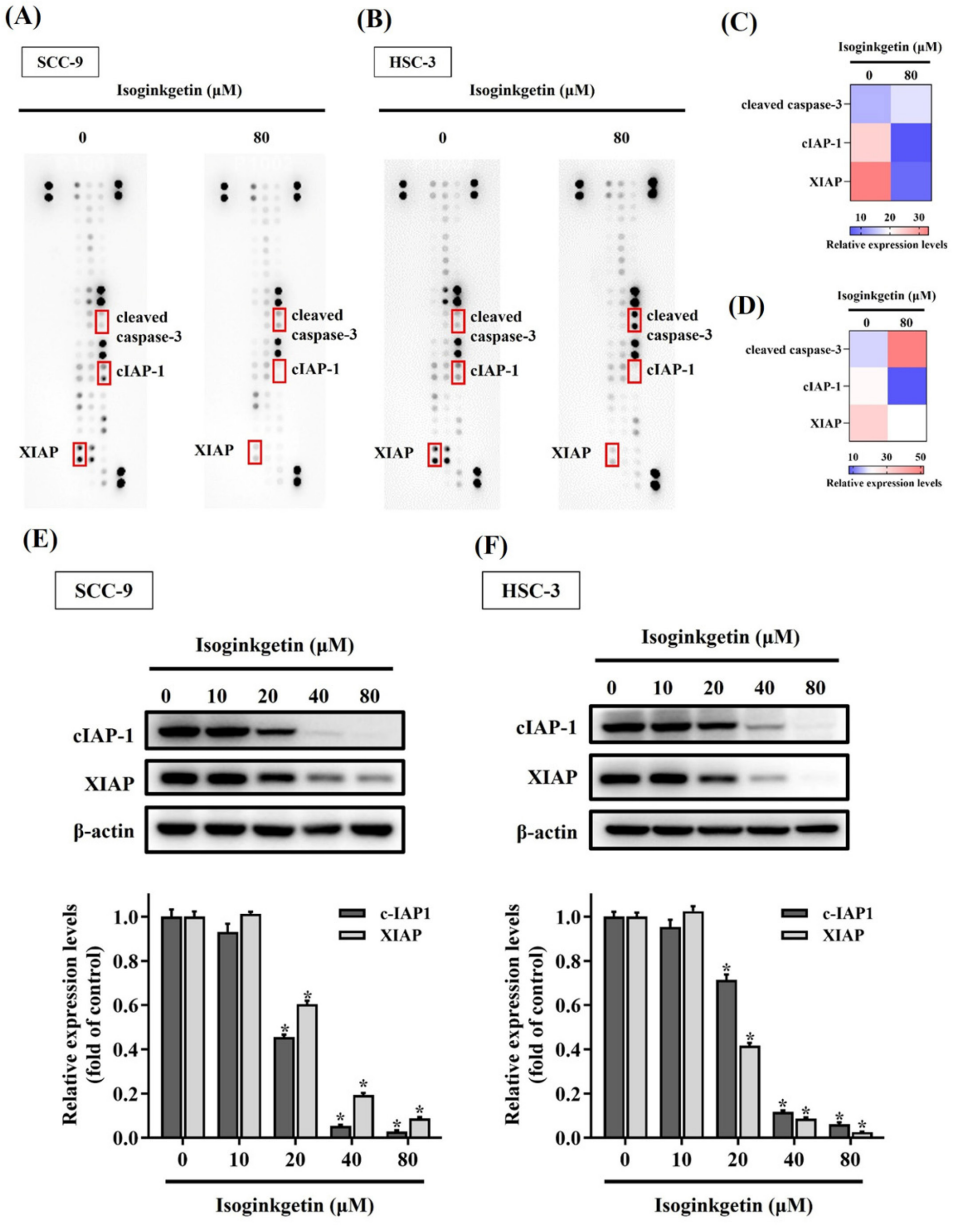
** Profiling of apoptosis-related proteins in IGG-treated OSCC cells.** (**A-B**) Representative dot plots corresponding to the levels of 35 apoptosis-related protein markers in IGG-untreated and -treated SCC-9 **(A)** and HSC-3 cells **(B)**. Markers with differential expression proteins are marked, labelled, and further verified. (**C-D**) Heatmaps depicting relative expression of selected dots from SCC-9 **(C)** and HSC-3 samples **(D)**. **(E-F)** Verification of apoptotic marker expression. SCC-9 **(E)** and HSC-3 cells** (F)** were maintained in the presence of indicated concentrations of IGG (10-80 μM) for 24 hr and assayed for the levels of indicated apoptosis markers by Western blot. Densitometric analyses of protein bands were conducted, normalized with internal controls (β-actin), compared, and shown underneath. Data represent the mean ± SD of three independent experiments. *p < 0.05, in comparison with untreated controls using Students t-test.

We subsequently aimed to investigate the profile of apoptosis-related proteins in IGG-treated OSCC cells by surveying 35 known protein markers of programmed cell death. A consistent shift in the expression levels of these markers was detected between two cell lines in response to IGG (**Figures 3A-D**). In particular, the levels of X-linked inhibitor of apoptosis protein (XIAP) and cellular inhibitor of apoptosis protein-1 (cIAP-1) in SCC-9 and HSC-3 cells were decreased under the treatment with IGG, whereas the signal intensities of cleaved caspase-3 were augmented in the same scenario. Further verification demonstrated that treatment with IGG downregulated XIAP and cIAP-1 in a dose-dependent manner (**Figures 3E-F**). These results indicate that IGG attunes the apoptotic proteome in OSCC cells, as manifested by downregulation of tentative apoptosis inhibitors.

### IGG promotes proteolytic cleavage of caspase substrates in OSCC

**Figure 4:**
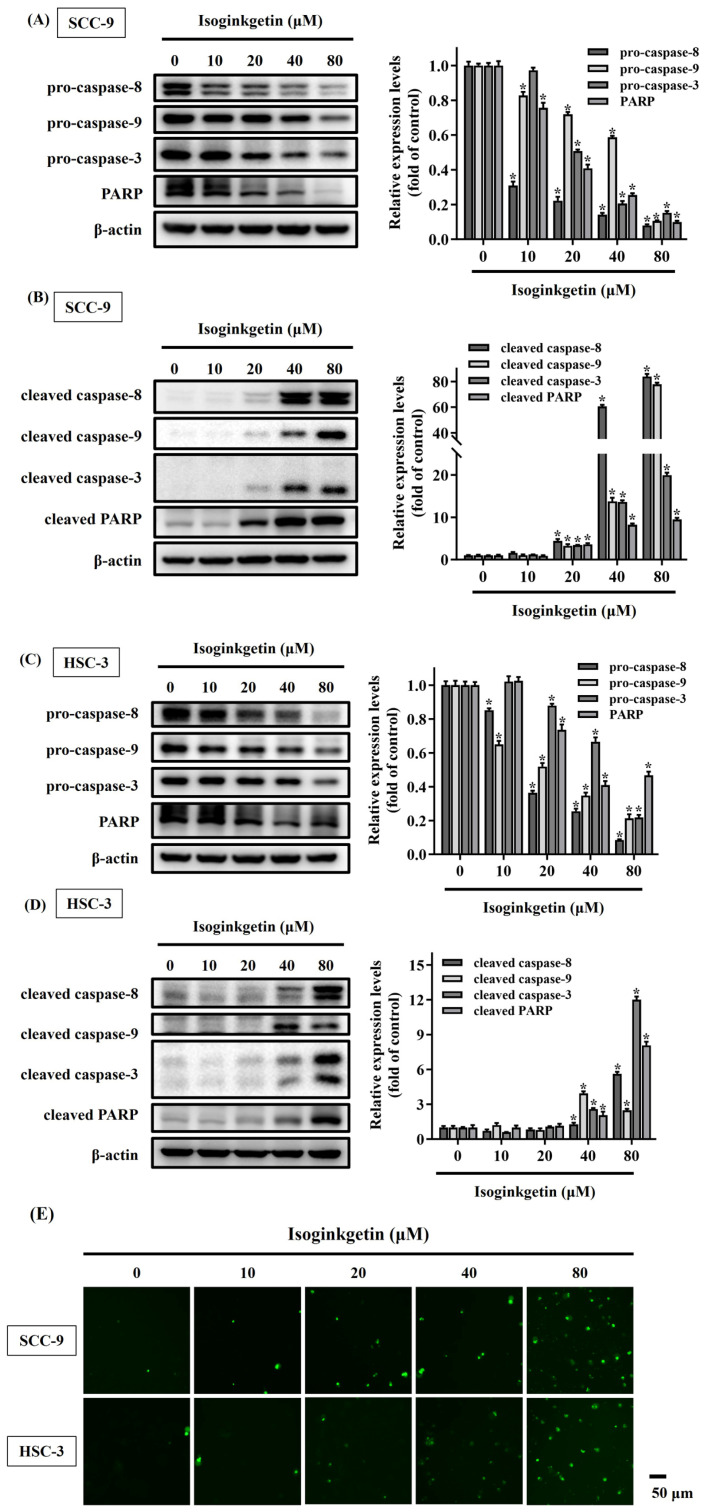
** Effect of IGG on promoting proteolytic cleavage of caspase substrates in OSCC.** SCC-9 and HSC-3 cells were maintained in the presence of indicated concentrations of IGG (10-80 μM) for 24 hr and assayed for the levels of precursor (**A, C**) and cleaved forms (**B, D**) of individual caspase substrates by Western blot. Densitometric analyses and signal comparisons are shown in the right. Data represent the mean ± SD of three separate experiments. *p < 0.05, compared with untreated controls using Students t-test. **(E)** Representative immunofluorescence images of cleaved caspase-3 in IGG-treated OSCCs. SCC-9 and HSC-3 cells were treated with IGG and stained with an antibody against cleaved forms of caspase-3. Bar = 50 μm.

Cleavage of caspase substrates is a hallmark of cell apoptosis that generates many forms of active fragments to mediate cell membrane blebbing, cell body shrinkage, and DNA fragmentation 20. Therefore, the effect of IGG on the cleavage of caspase substrates was explored in OSCC cells. We found that treatment of SCC-9 and HSC-3 cells with IGG, especially at 40 and 80 μM, reduced the levels of precursor (inactive) forms of caspase-3, -8, -9 and poly (ADP-ribose) polymerase-1 (PARP), accompanied with increased production of cleaved (active) forms of these apoptotic mediators in both lines (**Figures 4A-D**). In addition, activation of caspase 3 by IGG was further validated by immunofluorescence labeling of cleaved caspase 3 in SCC-9 and HSC-3 cells treated with IGG at different concentrations (**Figure 4E**). These results further support the observation that IGG acts as an inducer of apoptotic cell death in oral malignancy.

### JNK contributes to IGG-stimulated caspase activations in OSCC

**Figure 5:**
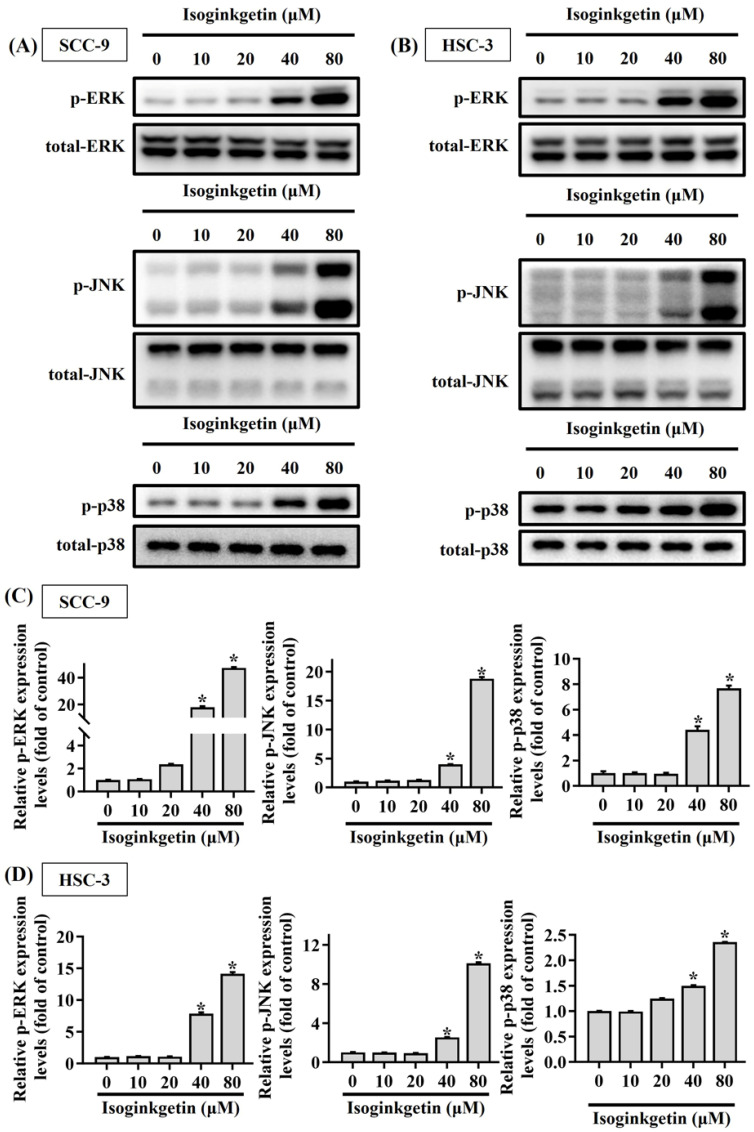
** Promotive effect of IGG on MAPK phosphorylation in OSCC cells.** SCC-9 **(A)** and HSC-3 cells **(B)** were incubated with IGG at indicated concentrations and tested for the levels of phosphorylation on individual MAPKs via Western blot. **(C-D)** Quantification and comparison of relative phosphorylation status for ERK1/2 (ERK), JNK, and p38-MAPK in each condition of SCC-9 **(C)** and HSC-3 cells **(D)**. The values represent the mean ± SD of three independent experiments. *p < 0.05, compared with untreated controls using Students t-test.

**Figure 6:**
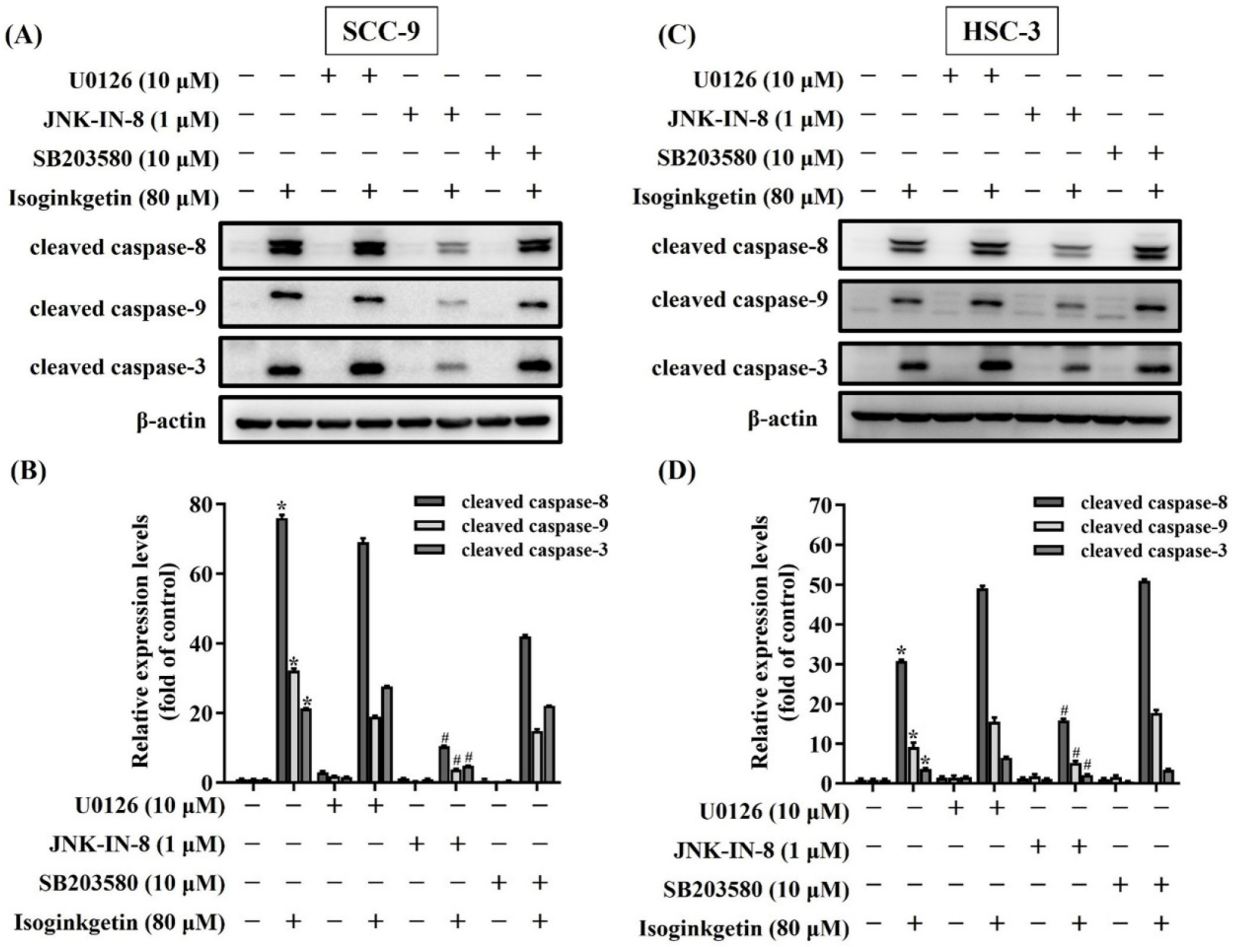
**Functional connection of JNK to IGG-activated caspase cascades in OSCC.** SCC-9 (**A-B**) and HSC-3 cells **(C-D)** were pretreated with individual MAPK antagonists for 2 hr and subsequently incubated with IGG for 24 hr, followed by assessment for the degree of caspase cleavage via Western blot. Densitometric analyses of SCC-9 **(B)** and HSC-3 data **(D)** were conducted, and relative expression levels were normalized to internal controls (β-actin). Data represent the mean ± SD of three separate experiments. *p < 0.05, compared with untreated controls using Students t-test. #*p <* 0.05, compared with IGG-treated cells using Students t-test.

Mitogen-activated protein kinases (MAPKs) are known to function as a key regulator to direct apoptotic responses to various external stresses 21-23. In the context of OSCC, dysregulation of MAPK signaling has been implicated in tumor progression and resistance to therapy 21, 24, 25. We next tried to dissect whether MAPKs are functionally involved in the activation of caspase cascades in IGG-treated OSCC cells. Our survey of MAPK phosphorylation status revealed that JNK, ERK, and p38-MAPK were highly phosphorylated in both OSCC cell lines as treated with 40 and 80 μM IGG (**Figures 5A-D**), indicating a promotive effect of IGG on MAPKs activation in oral cancer. To further unravel whether there is a functional link between MAPK phosphorylation and caspase activation in the process of IGG-induced apoptosis, we tested the influence of kinase inhibitions on caspase cleavages in IGG-treated OSCC cells. Our results demonstrated that blockage of JNK activation with JNK-IN-8, a specific JNK inhibitor, significantly diminished the induction of cleaved (active) caspase-3, caspase-8, and caspase-9 in IGG-treated SCC-9 and HSC-3 cells (**Figures 6A-D**). Yet, pharmaceutical inhibition of ERK and p38-MAPK did not affect IGG-induced cleavage of pro-caspase-3, -8, and -9 in both cell lines. These findings connect JNK activity with activation of caspase cascades during IGG-stimulated oral cancer cell apoptosis.

## Discussion

**Figure 7:**
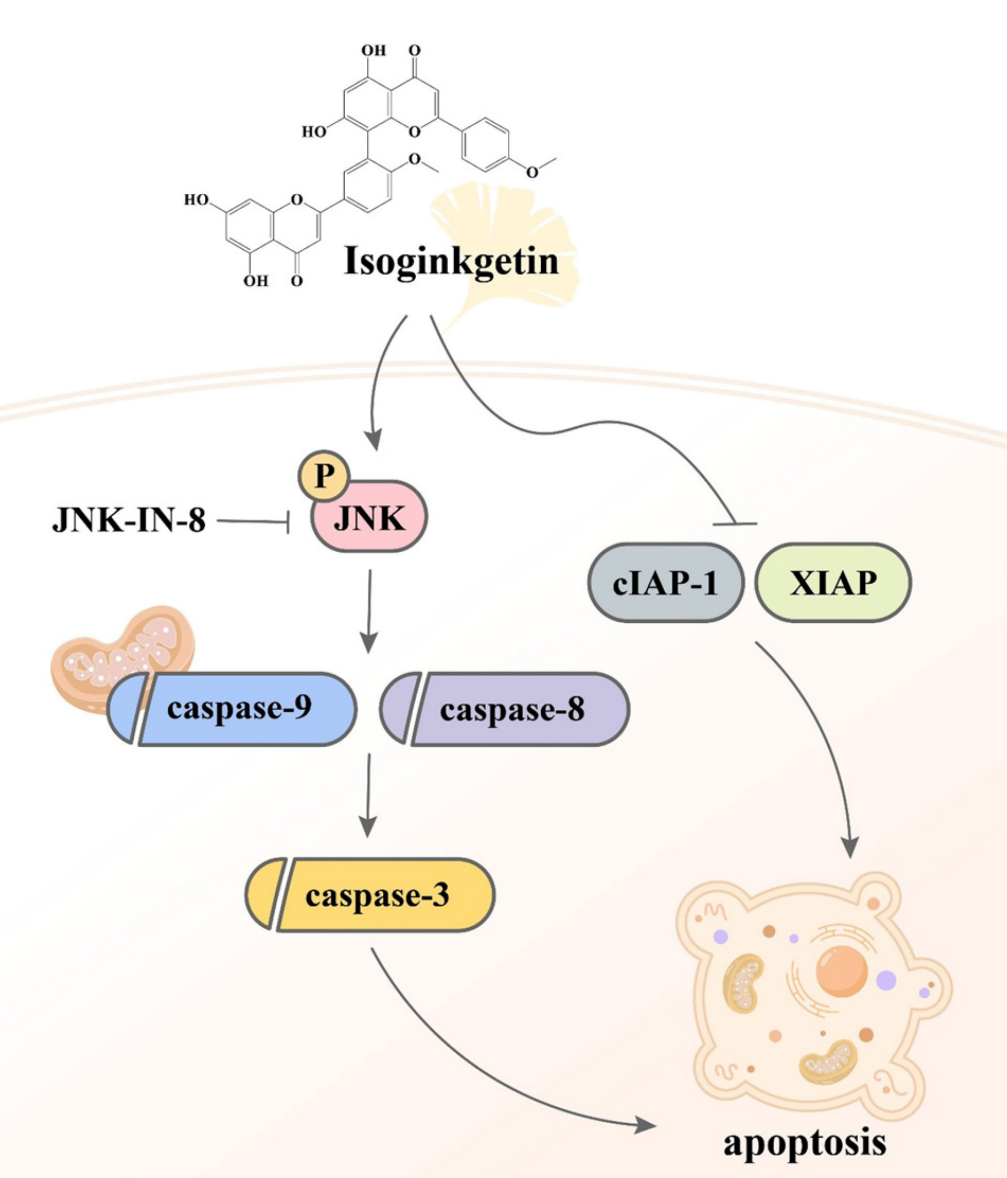
**Proposed mechanism of IGG-induced cell apoptosis in OSCC**.

Despite the observations that contemporary therapeutic methods have generated favorable outcomes in patients with early-stage oral malignancy, the survival rate and prognostic response of patients bearing late-stage OSCC still present an enormous burden. The application of complementary treatment options, thus, is needed to deal with the clinical challenge. It is widely accepted that natural constituents isolated from herbal plants can be beneficial for cancer treatment as given in combination with other standard cares 26. In our investigation, we demonstrated that IGG, a naturally occurring biflavonoid found in the leaves of ginkgo (*Ginkgo biloba*), effectively induced apoptotic responses in OSCC cell lines. Furthermore, the molecular mechanism underlying IGG-stimulated cell apoptosis involves downregulation of several apoptotic inhibitors and a JNK-dependent activation of caspase pathways (**Figure 7**). Our results highlight a potential of IGG in improving the management of OSCC.

Several studies of phytomedicine have documented a tumor-suppressive effect of IGG on various malignant diseases 7, 10, 13, 14, 27-29. Such anti-cancer activity relies upon multiple cell type-specific or general mechanisms and affects a variety of molecular targets. Firstly, IGG is a general inhibitor of spliceosome to block pre-mRNA splicing, thereby interfering with tumor growth 10. Unlike other anti-cancer natural compounds (e.g. pladienolide and spliceostatin A) that target the splicing factor SF3b to prevent the assembly of prespliceosome (A complex) 30, 31, the presence of IGG promotes accumulation of A complex and mediates its progression into B complex, a catalytically active form of spliceosome 10. Although these spliceosome inhibitors have distinct core structures and affect different steps of splicing, disruption of mRNA splicing leads to generation of aberrant proteins and triggers apoptotic cell death 32, 33. These findings are in accordance with our data showing a promotive effect of IGG on apoptotic responses in OSCC. Intriguingly, these spliceosome-targeted agents elicit double-stranded RNA responses via induction of widespread intron-retained transcripts to drive not only extrinsic apoptosis but also adaptive immune signaling in fighting cancer 34. Consistently, inhibition of the splicing machinery by IGG in malignant cells augmented the antigen presentation by MHC class I 11, thereby boosting the downstream adaptive immune response against tumor antigens 12. In addition to mRNA processing, IGG was also reported to inhibit transcription elongation, further highlighting its impact on regulation of gene expression 9. Moreover, IGG has been shown to sensitize cancer cells to apoptosis via impairment of protein clearance through directly inhibiting activities of 20S proteosome 13. The 20S proteosome simply degrades unfolded or misfolded proteins to maintain proper protein dynamics, and its inhibition could stimulate the unfolded protein response (UPR) 35, a signal transduction pathway that is activated by accumulation of excessive unfolded proteins and eventually renders the vulnerability of malignant cells to death 36. These findings, together with our data, implicate the use of IGG as a promising OSCC therapy.

Beside caspase activation, IGG-induced OSCC apoptosis was accompanied with downregulation of several putative apoptosis inhibitors. One such example is XIAP (X-linked inhibitor of apoptosis protein), an E3 ubiquitin ligase that has been shown to promote resistance to therapy-induced apoptosis and confer poor outcome in cancer patients 37. Different biological functions of XIAP localized at distinct cellular compartments, such as cytoplasm, mitochondria, and nucleus, have been documented. Cytoplasmic XIAP can physically interact with caspase-3, -7, and -9 and subsequently restrain activation of these caspases to counteract apoptosis 38, 39, whereas nuclear XIAP acts to modulate many oncogenic pathways 40-42, irrelevant to its caspase-inhibitory cytoplasmic function. Although cellular localization of XIAP was not investigated in our experiments, our observation that IGG downregulated XIAP and activated caspase cascades during OSCC apoptosis is largely in agreement with the anti-apoptotic function of cytotoxic XIAP. In addition, another IGG-downregulated proteins, cIAP-1 (cellular inhibitor of apoptosis protein 1), is also an E3 ubiquitin ligase that has a key role in regulating NF-*κ*B signaling and programmed cell death via the ubiquitylation of major components of TNF receptor complexes 43. cIAP-1 is a substrate for caspase-8, and its degradation by caspase-8 is associated with TNF-related apoptosis 44. Furthermore, cIAP-1 binds to the apoptosome and sterically hinders the access of pro-caspase-3 to the catalytic center of the Apaf-1-caspase-9 complex, thereby inhibiting the processing and activation of pro-caspase-3 45. These IGG-downregulated apoptosis mediators appear to act upstream or downstream of multiple caspase cascades, cooperatively contributing to IGG-stimulated apoptotic events in OSCC.

JNK signaling is known to mediate the extrinsic apoptotic pathway initiated by death receptors as well as the intrinsic pathway initiated at the mitochondria 46. In our study, IGG triggered apoptotic responses in OSCC, employing a JNK-dependent activation of caspase cascades. Consistently, such involvement of JNK activities in phytomedicine-induced caspase activation and apoptosis was observed in oral cancer 47, 48 and other cancer types 49-51. Our findings reiterate that JNK signaling behaves as a critical hub in caspase-dependent apoptosis of IGG-treated oral cancer cells.

Even though anti-cancer effects of IGG on oral carcinogenesis were observed, there are some limitations to this study. One weakness is that both OSCC cell lines tested in our experiments originated from tongue cancer. It is proposed that cancers developed at distinct anatomical locations of the mouth (e.g. tongue, buccal mucosa, lip, and gingiva) tends to be correlated with different mutational signatures, oncogenic pathways, and survival rates 52. Thus, the generalizability of this study can be reinforced if additional OSCC cell lines derived from other anatomical positions are examined to explore IGG's actions. Another concern is that the influence of this biflavonoid compound might be incongruous in animal studies, despite our *in vitro* finding that IGG rendered a tumor-suppressive effect on oral cancer progression. As IGG has exhibited promising oncostatic characteristics in mouse cancer models 12, 14, future experiments in *in vivo* settings could further clarify the impact of IGG on oral carcinogenesis.

In conclusion, we showed that IGG effectively triggered apoptotic cell death in OSCC, employing a JNK-dependent pathway of caspase activations. Our findings implicate this natural compound as a tentative therapeutic modality against oral malignancies.
